# What goes in a funder’s Narrative CV?: A Scoping Review

**DOI:** 10.12688/f1000research.170507.1

**Published:** 2025-12-01

**Authors:** Marc Antonino Albert, Aleeza Qayyum, Kailyn MacKinnon, Janina Ramos, Gustavo de Paula Dídimo, Gabriela Ferreira Kalkmann, Anna Catharina Vieira Armond, David Moher, Kelly D Cobey

**Affiliations:** 1 Metaresearch and Open Science Program, University of Ottawa Heart Institute, Ottawa, Ontario, K1Y 4W7, Canada; 2 University of Ottawa Faculty of Medicine, Ottawa, Ontario, K1H 8M5, Canada; 3 School of Epidemiology and Public Health, University of Ottawa Faculty of Medicine, Ottawa, Ontario, K1H 8M5, Canada; 4 Centre for Journalology, Ottawa Hospital Research Institute, Ottawa, Ontario, K1Y 4E9, Canada

**Keywords:** Narrative Curriculum Vitae, Research Integrity, Research Funders, Research Assessment, Research integrity, Science funding, Research ethics, Responsible Conduct of Research, Science communication, Research environment

## Abstract

Narrative Curriculum Vitae (NCVs) are a type of CV focusing on written descriptions of researchers’ skills, experiences, collaborations, and achievements, which seek to promote more equitable and responsible research assessments. Despite an apparent shift by funding organizations towards the use of NCVs to reassess how researchers are evaluated, the extent of current NCV adoption is unclear. Therefore, we conducted a scoping review to answer the following: 1. Which research funding agencies currently provide NCVs templates for their applicants? and 2. What are the characteristics of said NCV templates? To this end, we employed grey literature searches to identify all existing NCV templates provided by research funding organizations. Our findings highlight several key insights. First, number of funders currently requiring NCVs remains low overall, although national granting agencies are among the early adopters. Second, some funders do not provide formal guidance on how to complete narrative CV’s—this may create barriers to uptake. Third, among the NCV templates identified, there are structural commonalities, although there is little insight into the evaluation of NCVs. As interest in NCVs grows, addressing these gaps will be essential to realizing their potential as a fairer and more holistic tool for research assessment.

## Introduction

Narrative Curriculum Vitae (NCVs) are a type of CV with a focus on written descriptions of a researcher’s skills, experiences, collaborations, and achievements across a broad range of research activities. ^1^ NCVs seek to promote more equitable and responsible research assessment for funding, awards, grants, and hiring and promotion decisions, as compared to traditional academic CVs, ^2^ which are typically heavily dependent of quantitative metrics (e.g., number of publications). Early adopters of NCVs include The Dutch Research Council, ^3^ the Health Research Board Ireland, ^4^ the Luxembourg National Research Fund, ^5^ the National Institutes of Health in the United States, ^6^ Science Foundation Ireland, ^7^ the Swiss National Science Foundation, ^8^ and UK Research and Innovation. ^9^ A shift by funding organizations towards the use of NCVs is part of a broader global effort to reassess how research and researchers are evaluated, aiming to better align research incentives with mission-driven goals. ^10^ Initiatives such as the Declaration on Research Assessment (DORA), ^11^ the Leiden Manifesto, ^12^ and the Coalition for Advancing Research Assessment (CoARA) ^13^ have been pivotal in raising awareness of the shortcomings of reducing research (er) assessment to quantitative metrics. For example, researchers have traditionally been evaluated based on quantitative metrics such as the number of publications they produce, and the prestige, or “impact factor”, of the journals in which their research is published. ^14, 15^ These metrics are heavily impacted by factors like geographical location, ^16^ significance of the results, ^17^ study type, ^17^ funding and other declarations, ^17^ and reputation of the researcher’s institution. ^14^ This creates biases that perpetuate inequities by favouring established researchers and significant results ^17^ over emerging scholars and innovative ideas with less resources. ^18^ Moreover, the biases can decrease the diversity of research endeavours, which makes it more difficult for researchers to move between institutions, disciplines, or roles and stifles creativity. ^19^ Lastly, the biases from quantitative metrics can distort research priorities by shifting the focus away from meaningful scientific contributions onto high publication volumes and significant results.

NCVs offer the ability to reform research assessment by shifting the focus away from numbers and putting the power back in the hands or researchers to reflect qualitatively on what they have done and what its impacts have been. NCVs permit assessments centered around the research subject; the broad skills, competencies and experiences of researchers; the contributions of researchers to the dissemination of knowledge and innovation of research ^4^; and the rigor behind the research. ^10^ When used by funding organizations, NCVs can provide a more holistic overview of grant applicants’ academic and community contributions – leadership and mentorship experience, broader societal impacts. When compared to traditional CVs, NCVs also provide grant applicants with more flexibility regarding how they described their listed items, thereby allowing applicants to tailor their descriptions to each individual grant and better showcase their potential for meaningful impacts. ^20^

Despite their numerous benefits, certain limitations to NCVs have also been noted. Completing unique and detailed NCVs for distinct funding calls may increase the workload for grant applicants. ^19^ Additionally, concerns have been raised regarding how to complete NCVs and especially with respect to how they will be evaluated. This introduces subjectivity, which can lead to varied interpretations and hence inconsistent evaluations of researcher contributions, ^21^ thus creating confusion and undermining the credibility of the NCV approach. ^21, 22^ Some funding organizations have tried to address these challenges associated with a lack of clarity around NCVs by providing grant applicants with guidance or instruction, often in the form of NCV templates. The objective of this is to remove some of the subjectivity ingrained in the NCV approach, while retaining its benefits, facilitating applicants’ ability to effectively highlight their contributions and express their significance. However, continued efforts to improve upon NCV implementation for research funding requires a better understanding of the current breadth of NCV template used by funding organizations, as well as a mapping of the said templates’ characteristics.

## Objectives

Our objectives for this scoping review were to answer the following research questions: 1. Which research funding agencies currently provide NCV templates as part of their grant application process? and 2. What are the characteristics of NCV templates provided by funding agencies?

## Methods

***Transparency statement.*** The protocol for this study was registered on the Open Science Framework (OSF) ^23^ a priori; all study data, and a pre-print of this manuscript are also available on OSF (DOI: 10.17605/OSF.IO/4DGBR). This manuscript is reported in adherence to the PRISMA-ScR checklist for scoping reviews ^24^ (refer to supplemental materials). For details on our data management plan, see Appendix 1.

### Scoping review approach

Following the guidance of Arksey and O’Malley ^25^ and Levac and colleagues, ^26^ our scoping review consisted of five distinct steps: 1) Identifying the research question, 2) Identifying NCV templates, 3) Selecting the templates, 4) Charting the data, and 5) Analyzing, summarizing, and reporting the results.

*Step 1: Identifying the research question(s)*

In this scoping review, we aimed to answer the following questions: 1. Which research funding agencies currently require NCVs as part of their grant application process? and 2. What are the characteristics of NCV templates provided by said funding agencies?

*Step 2: Identifying NCV templates*

We did not conduct a traditional electronic search (e.g., Medline) because we felt that narrative CVs from funders were unlikely to appear in the traditional published literature.

To identify relevant NCV templates, we employed grey literature searches via two approaches. First, we conducted a grey literature search using *Google* to identify potential documents available outside of standard peer-reviewed literature. We piloted several search terms before settling on the following as our final search terms: “Narrative CV”, “Research CV Template”, and “Research Funder CV Template”. These selected search terms were intentionally broad to maximize the likelihood of capturing all available funder NCV templates. Each search was conducted by one individual and limited to documents reported in English. The full search strategy is described in Appendix 2.

Next, to identify any additional templates we used the website *healthresearchfunders.org* ^27^ to compile a list of research funders from across the globe, and then manually searched the websites of any funders for whom we did not identify an NCV template through our initial *Google* search. This manual search of funder websites involved using same three search terms. Acknowledging that this health researchers’ funders list has not recently been updated, we also supplemented this list based on our team’s knowledge of additional funding agencies and subsequently conducted manual searches of any additional funders’ websites using the same approach.

*Step 3: Selecting the NCV templates*

***Inclusion and exclusion criteria:*** We included all NCV templates utilized by research funders published in English. We did not restrict our search by date or year. We also included NCV templates that are not in current use but have been used in the past, and NCV templates that exist but have not yet been used in practice. We excluded NCV templates published in languages other than English, as well as any documents not publicly available online. Reasons for exclusion were noted for all excluded documents and are reported below in our results. These inclusion/exclusion criteria are outlined in Table 1.

**Table 1.** Eligibility criteria for research article to be included in sample for extraction and analysis.

Inclusion criteria	Exclusion criteria
- NCV template is in English - NCV template is publicly available online - NCV template is from a research funding organization or used for the purpose of granting a funding award	- NCV template is NOT in English - NCV template not publicly available online - NCV template is not from a research funding organization or is not used for the purpose of granting a funding award

***Selection/Screening:*** The Grey literature search was performed by two individuals (AQ & GdPD) who collated the citations using Zotero, ^28^ and subsequently added the identified documents into Covidence ^29^ for screening. The search based on the list from *healthresearchfunders.org* ^27^ was performed by one individual (AQ), who followed the same steps to aggregate the documents for screening. All documents were then independently screened against our inclusion criteria in duplicate; any conflicts were resolved via discussion between reviewers, and third-party arbitration when needed.

*Step 4: Charting the data*

We extracted data regarding several document characteristics in duplicate from all included NCV templates using Covidence. ^29^ The initial extraction was performed by one individual, while a second individual performed a quality-control review. We deductively created a purpose-built data extraction form ( ***Appendix 3)*** after conducting a preliminary scan of a few known NCV templates. The extraction form was piloted by three individuals prior to finalization to ensure its relevance; some minor modifications to the form were made throughout this process to improve clarity and completeness. For instance, despite planning to only collect initial dates of release for the templates, after noticing that multiple templates had undergone official updates, we modified our extraction form to also capture these update dates.

Extracted data included the name of the funding organization, the country of the funder, the date of the NCV template implementation, the number of questions and pages, and the name of the organization that created the NCV template. We also mapped the content of the NCV questions to gauge what topics were being asked about, and in what format responses were being captured. For qualitative data, we conducted a content analysis of the extracted text using Microsoft Excel. Without using preconceived categories, two researchers independently identified key themes in the data using inductive coding and then discussed these until consensus was achieved. The coded elements were re-incorporated into the dataset. Key themes were summarized and presented using a narrative synthesis with illustrative quotes or as counts and percentages reflecting the codes. All percentages presented in the text and tables were calculated using 21 NCV templates as the denominator, unless otherwise stated.

*Step 5: Report the results*

We have reported the results of this study by summarizing the features of the NCVs using descriptive statistics. These summarized features include both the explicit categories listed in our extraction form (for each item), as well as some additional features captured in our open-text extraction items, which we identified and organized via qualitative analysis.

**Protocol amendments**

We amended our initial protocol (registered on the OSF DOI: 10.17605/OSF.IO/4DGBR) by changing our qualitative analysis approach from Braun and Clarke’s thematic analysis to a content analysis. This decision was made because traditional thematic analysis (as described by Braun and Clarke) involves an in-depth analysis of themes and their meanings, ^30^ which we do not believe would be appropriate for the small amount of qualitative data collected via our few open-text extraction items. Thus, we modified our approach to perform a content analysis instead.

## Results

**Figure 1.**
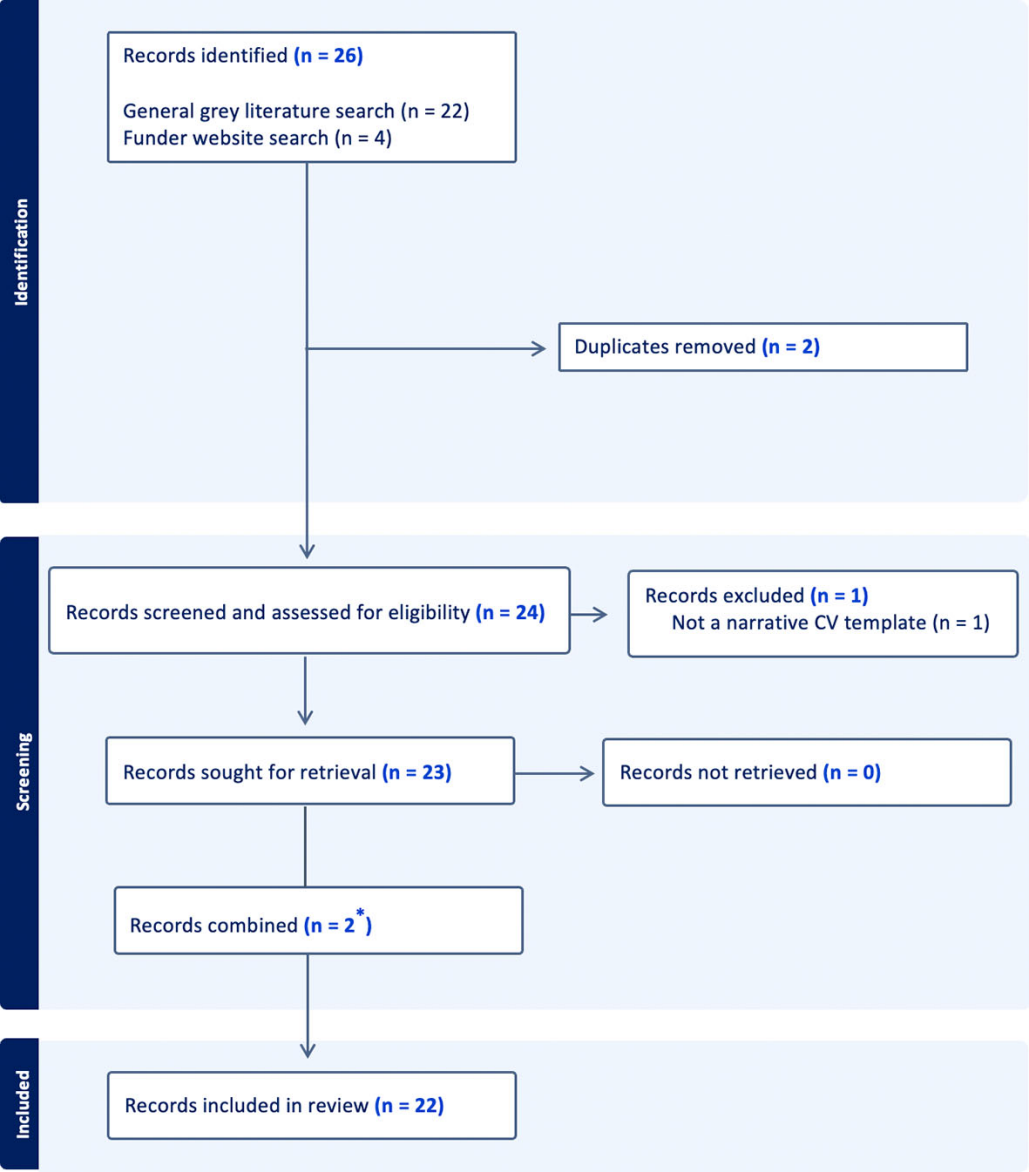
Flow diagram depicting inclusion/exclusion criteria for screening of NVC templates. The primary search conducted via a general grey literature search using pre-specified key search terms. These same search terms were then used manually search the websites of funders for whom our primary search did not identify any templates – which websites to search was based on a list of funders compiled using healthresearchfunders.org. ^27^ The flow diagram is adapted from the PRISMA Statement, 2009. ^32^ ^*^Two retrieved records were from the same funder (one template; one illustrative example) and thus were combined for data extraction.

The key findings of this scoping review are summarized below; however, all extracted data are available as supplemental material in CSV format. Our screening process is outlined in using a PRISMA flow diagram ^31, 32^ (refer to Figure 1).

### Demographic characteristics of funders with NCV templates

In total, we extracted and analyzed data from 22 NCV templates, each from a different funding organization, originating from 13 different countries: the United Kingdom (n = 5), New Zealand (n = 2), Switzerland (n = 2), Canada (n = 2), the United States (n = 2), Ireland (n = 2), Norway (n = 1), the Netherlands (n = 1), Germany (n = 1), Belgium (n = 1), Brazil (n = 1), Finland (n = 1), and Luxembourg (n = 1). The templates belonged to three types of funders: National Granting Agencies (n = 17, 77%), Foundations/Philanthropic Bodies (n = 4, 18%), and a Hospital Research Institute (n = 1, 5%). The funders pertained to several research disciplines (some to multiple disciplines): Medicine/Biomedical Sciences (n = 10, 45%), Natural Sciences (n = 3, 14%), Social Sciences and Humanities (n = 2, 9%), Business and Economic Development (n = 1, 5%), and Arts (n = 1, 5%). Additionally, nine funders (41%) were discipline agnostic. These demographic characteristics of the funders to whom the NCV templates belong, as well as the names of the funders, are outlined in Table 2.

**Table 2.** Demographic characteristics of research funders with NCV templates for grant applications (N = 22).

	Count (n, %)
**Type of funding organization**	
National Granting Agency	17 (77%)
Foundation/Philanthropic Body	4 (18%)
Hospital Research Institute	1 (5%)
**Discipline of funding organization**	
Medicine/Biomedical Sciences	10 (45%)
Natural Sciences	3 (14%)
Social Sciences and Humanities	2 (9%)
Business and Economic Development	1 (5%)
Arts	1 (5%)
Discipline agnostic	9 (41%)
**Country of origin and name of funding organizations**	
**United Kingdom**	5 (23%)
- Medical Research Foundation - Alzheimer’s Research UK - The Royal Society - Cancer Research UK - UK Research and Innovation (UKRI)	
** New Zealand**	2 (9%)
- Health Research Council of New Zealand - Ministry of Business, Innovation & Employment (MBIE) ^1^	
**Switzerland**	2 (9%)
- Swiss National Science Foundation (SNSF)	
** Canada**	2 (9%)
- Tri-Agency ^2^ - Canada Council for the Arts	
**United States**	2 (9%)
- National Institutes of Health (NIH) - Massachusetts General Hospital	
**Ireland**	2 (9%)
- Science Foundation Ireland (SFI)	
- Health Research Board (HRB)	
**Norway**	1 (5%)
- The Research Council of Norway	
**The Netherlands**	1 (5%)
- Dutch Research Council (NWO)	
**Germany**	1 (5%)
- Deutsche Forschungsgemeinschaft (DFG)	
**Belgium**	1 (5%)
- Fonds voor Wetenschappelijk Onderzoek - Vlaanderen (FWO)	
**Brazil**	1 (5%)
- Fundação de Amparo à Pesquisa do Estado de São Paulo (FAPESP)	
**Finland**	1 (5%)
- Research Council of Finland	
**Luxembourg**	1 (5%)
- Fonds National de la Recherche Luxembourg (FNR)	

All percentages are rounded to the nearest integer.

^1^ Two documents were retrieved from this organization (NCV template + Illustrative example of completed NCV).

^2^ The Tri-Agency of Canada consists of three granting agencies: the Canadian Institutes of Health Research (CIHR), the National Sciences. and Engineering Research Council of Canada (NSERC), and the Social Sciences and Humanities Research Council of Canada (SSHRC).

The templates were introduced over a wide range of years (range: 2012-2024), with the most common year of introduction being 2022 (n = 3, 14%). Some templates (n = 5, 23%) have been updated since their initial release, with the longest period between initial release and official update being from the Research Council of Finland’s template, which was updated in 2020 – eight years after its initial release. There were also 10 templates (45%) for which the year of introduction was unclear. All 22 NCV templates are currently being used by their respective organizations.

### Development and structure of NCVs

**Figure 2.**
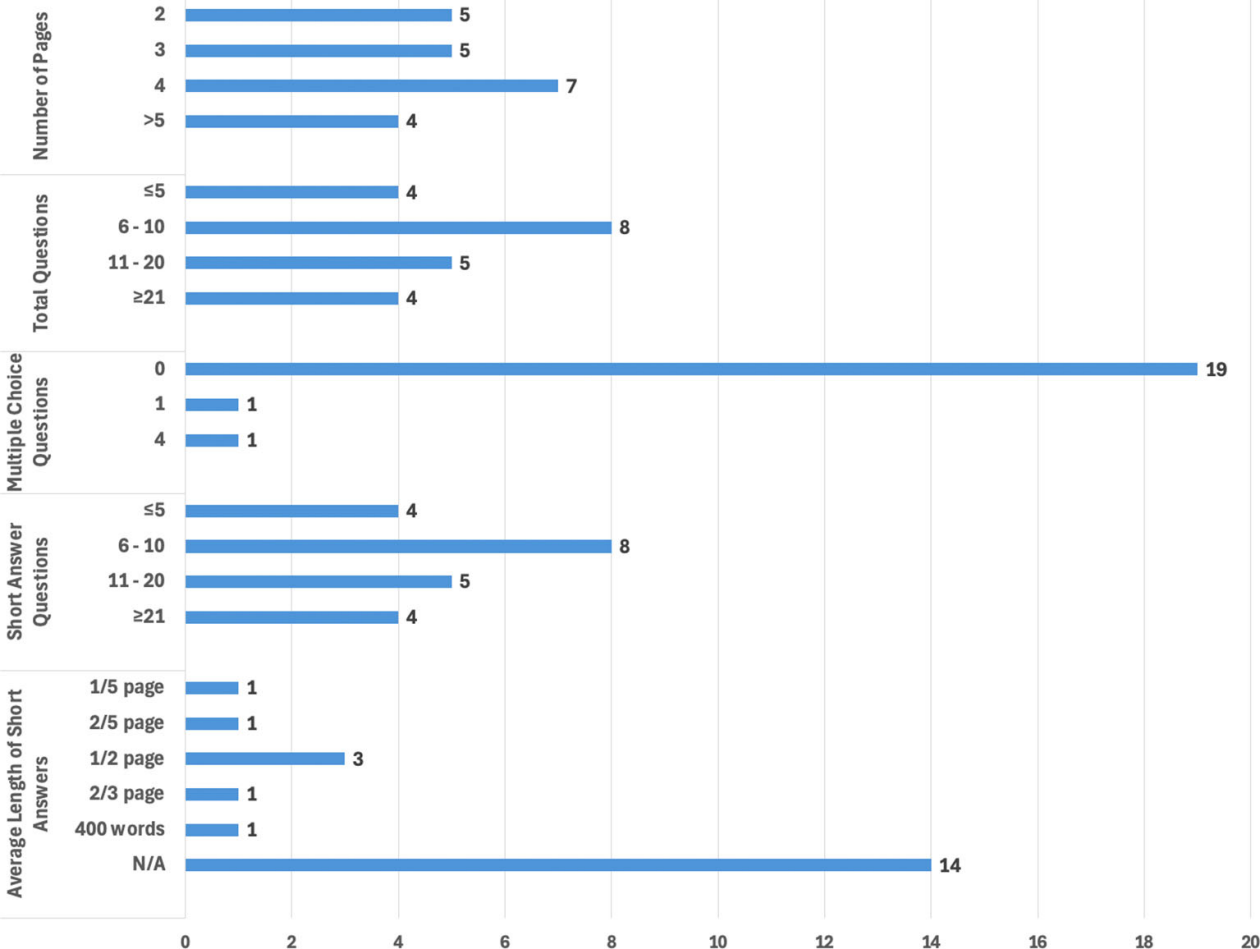
Bar graph depicting the distribution of NCV templates by length, and number and type of questions (N=22). “Average Length of Short Answers” refers to the recommend average length of each short answer item within the template. Applicant information to be included when completing the NCVs.

We also extracted several data characteristics regarding structure and content (e.g., the number, type, and subject matter of the questions listed) of each NCVs to map what information is being requested of grant applicants by funders. NCVs varied in length with respect to the number of pages (range: 2-9 pages; median = 4 pages). Similarly, the total number of questions/items in the templates varied widely (range: 3-31 questions; median = 8.5 questions). Most NCV templates did not contain any multiple-choice questions (n = 19, 86%), but the three templates which did, contained one, one, and four multiple choice questions, respectively. Conversely, all templates included short answer questions; the distribution of which was nearly identical to the total number of questions (range: 3-31 short answer questions; median = 8 short answer questions). Regarding these short answer items, most templates did not indicate a desired response length (n = 14, 64%), but a few did indicate average expected response lengths (range: 1/5 page- 400 words/question; mode: 1/2 page per question). The length and question distribution of the NCV templates is depicted in Figure 2.

The NCV templates requested a range of information from prospective grant applicants, all of which is outlined in Table 3. Most templates included general questions about applicants’ journal publications (n = 18, 82%), career breaks or major life events (n = 17, 77%), relevant areas of expertise (n = 16, 73%), academic record/educational qualifications (n = 16, 73%), career stage (n = 13, 59%), and awards & prizes (n = 13, 59%). Additionally, some templates collected information regarding other grant funding received (n = 10, 45%) or conference presentations given (n = 8, 36%). Some templates also asked applicants to provide a personal statement (n = 8, 36%), or a description of how they were impacted by the COVID-19 pandemic (n = 4, 18%).

**Table 3.** Overview of the questions asked, and information collected in NCV templates to be submitted as part of grant applications for research funding (N = 22).

Item	Count (n, %)
**General information**	
Information about journal publications	18 (82%)
Career breaks or major life events	17 (77%)
Relevant areas of expertise	16 (73%)
Academic/educational qualifications	16 (73%)
Current career stage	13 (59%)
Awards/Prizes	13 (59%)
Grant funding received	10 (45%)
Conference presentations given	8 (36%)
Personal statement	8 (36%)
Impact of the COVID-19 pandemic	4 (18%)
**Journal publications section**	
A general request to provide publication information	9 (41%)
Specific request for a selected number of important publications ^1^	9 (41%)
A requirement to list one’s total number of publications	3 (14%)
A requirement to indicate the peer-reviewed status of publications described	3 (14%)
A requirement to specify the open access status of each publication	2 (9%)
A requirement to outline one’s full list of publications	1 (5%)
A requirement to provide the Digital Object Identifier (DOI) of publications	1 (5%)
A requirement to provide a link to publication(s)	1 (5%)
No journal publication-specific section ^2^	4 (18%)
**Other grant funding received section**	
A general request for achievements	4 (18%)
A requirement to indicate the position/role on grants listed	2 (9%)
A requirement to specify the amount(s) awarded	2 (9%)
A requirement to list all research grants	1 (5%)
A requirement for a detailed description of a selected number of grants	1 (5%)
A request to list a selected number of significant research grants	1 (5%)
N/A	12 (55%)
**Contributions to the generation of new ideas/knowledge**	
New/Innovative tools, software & instruments	12 (55%)
New/Innovative ideas and hypotheses	11 (50%)
Patents & designs	10 (45%)
N/A	7 (32%)
**Teaching/mentoring contributions**	
Supervision skills/mentoring (e.g., trainees, students, staff )	14 (64%)
Teaching responsibilities (e.g., course(s), class (es), workshop(s))	12 (55%)
Teaching achievements/awards	5 (23%)
N/A	7 (32%)
**Contributions to open science**	
Knowledge mobilization/translation	8 (36%)
Data sharing	7 (32%)
Preprints	4 (18%)
Open access publishing	1 (5%)
Open educational resources	1 (5%)
General open science	1 (5%)
Open research	1 (5%)
Open source	1 (5%)
Open Science Initiatives	1 (5%)
No mention of open science	11 (50%)
**Contributions to the scientific/research community**	
Committee membership (e.g. boards, editorial roles, working groups)	15 (68%)
Leadership activities	12 (55%)
International involvement	12 (55%)
Editing or reviewing responsibilities	12 (55%)
Activities contributing to research integrity	10 (45%)
Activities contributing to equity, diversity and inclusion practices	9 (41%)
Event planning (conferences and workshops)	7 (32%)
N/A	5 (23%)
**Contributions to broader society**	
Partnerships with industry, healthcare or businesses	11 (50%)
Patient/public engagement	10 (45%)
Engagement with the media	9 (41%)
Involvement with policy development or public understanding	7 (32%)
Impacts on businesses	5 (23%)
General societal impact(s)	4 (18%)
Indigenous engagement	2 (9%)
Social impact	2 (9%)
Commercial impact	1 (5%)
Environmental impact	1 (5%)
General cultural impact	1 (5%)
Research culture impact	1 (5%)
**Does the NCV template include a section for ‘Additional Information’?**	
No	14 (64%)
Yes	8 (36%)
**Additional information specifically requested but not asked for in previous sections**	
ORCID or another unique researcher identifier	6 (27%)
Any additional outputs/impacts	4 (18%)
Indigenous impacts/considerations	1 (5%)
Language skills	1 (5%)
Other skills	1 (5%)

All percentages are rounded to the nearest integer.

^1^ Refers to wording like “main”, “important”, or “relevant” publications; or “key outputs”, or wording like “publications in major national or international peer-reviewed journals”. It was often left up to the applicants to interpret and decide which were ‘important’.

^2^ These four templates still provided applicants with space to describe their publications, but did not have a dedicated “journal publication” section.

Among those templates that included a section specific to journal publications, nine (41%) simply contained general requests for outputs, whereas nine (41%) others asked applicants to outline a specific number of important publications – often referred to as “main”, “important” or “relevant” publications, “key outputs”, or “publications in major national or international peer-reviewed journals”. Three templates (14%) required applicants to provide their total number of publications, while one template (5%) required applicants to provide a complete list of their publications. Additionally, some templates asked applicants to indicate the peer-review status (n = 3, 14%) or open access status (n = 2, 9%) of their publications. One also generically asked for links to their listed publications, whereas another explicitly asked for the DOIs of their listed publications.

Among templates that collected information around other sources of grant funding received by prospective applicants, four (18%) contained general requests for achievements (i.e., listing other funding sources), whereas one explicitly asked applicants to list a specific number of significant research grants, and another asked for all other research grants to be listed. In addition, some templates asked for specific information pertaining to said other sources of funding, such as the applicant’s position/role on the grant (n = 2, 9%), or the specific dollar amounts that were awarded (n = 2, 9%). Two templates asked for a detailed description of a specific number of other awarded research grants.

Many templates asked for information regarding applicants’ various contributions to the academic or research landscape, including contributions to the generation of new knowledges or ideas, teaching or mentorship, open science efforts, the general research/scientific community, or to broader society. With respect to contributions to the generation of new knowledges or ideas, roughly half of the templates had questions specific to new/innovative tools, software, and instruments (n = 12, 55%); new/innovative ideas and hypotheses (n = 11, 50%); or patents and designs owned (n = 10, 45%). Regarding teaching or mentorship contributions, many templates asked about supervision skills or mentoring experience (e.g., trainees, students, staff ) (n = 14, 64%), teaching responsibilities (n = 12, 55%), or teaching achievements and awards (n = 5, 23%). While many templates did not mention open science at all (n = 11, 50%), some specifically asked about knowledge translation or mobilization efforts (n = 8, 36%), data sharing (n = 7, 32%), preprints (n = 4, 18%), or other open science aspects (n = 6, 27%). All but five templates asked about general contributions to the scientific research community (n = 17, 77%), with the most common items being committee membership (e.g., roles on editorial boards or working groups) (n = 15, 68%), general leadership activities (n = 12, 55%), international involvement (n = 12, 55%), or editing or reviewing responsibilities (n = 12, 55%). With respect to other general societal contributions, the most frequently requested were partnerships with industry, healthcare, or business (n = 11, 50%); patient or public engagement (n = 10, 45%); engagement with the media (n = 9, 41%); and involvement with policy development or public understanding (n = 7, 32%).

Finally, some templates specifically asked for additional information that was not captured in other sections of template, these included any additional outputs or impacts (n = 4, 18%); ORCIDs ^33^ or other unique researcher identifiers (n = 6, 27%); indigenous impacts or considerations (n = 1, 5%), language skills (n = 1, 5%), or ‘other’ skills (n = 1, 5%). Many of the NCV templates also included a section wherein applicants could provide additional information at their own discretion (n = 14, 64%).

### Guidance and instructions provided regarding completion of NCVs

In addition to investigating the structure and contents of the templates, we also explored whether any guidance or instruction was provided to grant applicants by the funders. A majority offered at least some guidance within their template regarding how to complete the NCV (n = 12, 55%), whereas a slight minority (n = 10, 45%) provided similar guidance online (i.e., on the funders’ website). Guidance was offered in several forms: a total page limit (n = 9, 41%), section headers with detailed step-by-step instructions and examples (n = 6, 27%), section headers with general instructions (n = 6, 27%), specific formatting instructions (n = 5, 23%), a total word limit (n = 1, 5%), and links to additional resources (n = 1, 5%). Additionally, two templates included comprehensive examples of how they should be completed (n = 2, 9%), one template mentioned that it was optional to use, another template denoted certain sections as optional to complete, and two templates specifically mentioned that they were adapted from another template or resource (n = 2, 9%). Finally, four (18%) of the templates were accompanied by a description of how the NCV would be appraised by reviewers. The body of guidance and instruction provided across our sample of NCV templates is outlined in Table 4.

**Table 4.** Overview of the guidance or instructions provided to grant applicants by funders regarding how to complete their NCV (N = 22).

		Count (n, %)
**Guidance provided in the template document**		
Yes		12 (55%)
No		10 (45%)
**Guidance provided online**		
Yes		10 (45%)
No		12 (55%)
**Type of guidance**	**Illustrative example**	
Total page limit	*“The final version must not exceed four pages”*	9 (41%)
Section headers with detailed description/instructions and examples	*“1b. Academic qualifications* - *Delete and start typing here.* - *List in reverse date order.* - *Start each qualification on a new line as per the example: e.g. Year conferred, qualification, discipline, university/institute…”*	6 (27%)
Section headers with general instructions	*“Section 5 – Societal Impact* - *Contributions towards wider social benefit/impact which may include collaboration with communities, organizations, practitioners (‘research-users’), etc. ….”*	6 (27%)
Specific formatting instructions	*“This document should be completed in Arial 11pt.”*	5 (23%)
Links to additional resources	*“The University of Glasgow has created an online resource giving guidance on filling out narrative-style CVs. Here is the link to the website and accompanying videos:”*	1 (5%)
Total word limit	*“Total word limit section 1 + section 2: 1200 words”*	1 (5%)
Specific section optional	*“Optional Personal Statement:* - *You have the opportunity here if you wish, to provide a personal statement that reflects on your overarching past, present, and future professional goals.”*	1 (5%)
Whole template optional	*“You can use this optional template to build your CV, or you can use one you already have”*	1 (5%)
**Description of appraisal process**		
Yes	*“Applicants should be advised that evidence and examples provided in sections 1-4 will form a significant part of the assessment of applications”*	4 (18%) ^1^
No	N/A	18 (82%)

All percentages are rounded to the nearest integer.

^1^ Three funders described the NCV appraisal process within the template; one provided a description online.

## Discussion

This scoping review sought to address two key research questions: (1) Which research funding agencies currently require NCVs as part of their grant application process? and (2) What are the characteristics of the NCV templates used by these agencies? We identified 22 NCV templates from funders in 13 countries—a relatively modest number considering that healthresearchfunders.org alone lists 287 organizations across 30 countries. ^34^ Nevertheless, this group includes prominent national funders such as the Canadian Tri-Agency, UK Research and Innovation (UKRI), and the U.S. National Institutes of Health (NIH), suggesting that interest in NCVs is growing among key funders. We were a little surprised that few ‘qualitative’ disciplines promoted NCVs; medicine/biomedical sciences most frequently used NCVs (50%) and few philanthropies (18%) used NCVs.

The limited uptake of NCVs compounds a frequently raised concern: they add to applicant workload in an already demanding grant application process. ^19^ Unlike traditional CVs, NCVs require applicants to provide qualitative narratives and reflect on the impact of their work, making them more time-consuming to complete. This burden is exacerbated by the lack of standardization across NCV formats, requiring applicants to tailor their CVs differently for each funder. Additionally, 45% of the organizations requiring NCV provided no guidance as to how to populate them. This inefficiency is reminiscent of the academic publishing industry’s non-standardized formatting requirements, which are estimated to waste $200 million USD annually [36]. Just as initiatives have emerged to harmonize article submission processes, similar efforts could help streamline grant applications by promoting consistency in NCV requirements.

Despite these challenges, we observed encouraging consistency across many of the templates reviewed. Most NCVs requested similar types of information—such as journal publications, career breaks, research areas, educational background, awards, and career stages—and used predominantly short-answer questions. These commonalities suggest that funders broadly agree on the types of contextual information that are valuable for evaluating a researcher’s contributions. Some quantitative metrics were still included, but many templates reflected a shift toward more holistic assessments of scholarly impact, aligning with principles endorsed by DORA (the Declaration on Research Assessment). For instance, some funders maintained references to “high-impact journals,” but the overall trend favored narrative descriptions over purely bibliometric indicators. Additionally, the degree of instruction varied widely—from minimal guidance to highly detailed examples and completed templates. This variability may lead to confusion for applicants and inconsistencies in how reviewers interpret and assess NCVs.

A particularly concerning gap is the lack of transparency around the evaluation of NCVs. Only a few templates outlined how responses would be appraised by reviewers. This lack of clarity introduces subjectivity, which could undermine both the credibility and consistency of NCV-based assessments. ^21, 22^ Increasing transparency about the review evaluation process will help standardize expectations and build trust in the use of NCVs. It may be prudent to strike up an interest holder committee to address this important shortcoming.

To improve NCV adoption and effectiveness, we suggest several strategies. First, given the structural similarities already present, funders could collaborate to further harmonize NCV formats. While some variation is inevitable due to differing disciplinary norms and funder priorities, a coordinated approach would ease the burden on applicants and support broader uptake. Reporting guidelines have proven successful in harmonizing (across journals) ways to report specific types of study designs, such as randomized trials (CONSORT). Second, funders should prioritize providing robust guidance on how to complete NCVs, ideally including example responses. Third, and perhaps most importantly, funders must be more transparent about how NCVs are evaluated. Clear criteria and reviewer guidelines would go a long way toward ensuring fair and consistent assessments. We would also like to see an evaluation of the effectiveness of any training administered to peer reviewers to demonstrate effectiveness.

Several funders have now embraced open science and included mandates or recommendations about how this information should be included in grant applications, such as study registration, data management and sharing plans, and data deposits. Yet we found that 50% of funders had no requirements to report on any open science information, such as use of preprints or open access publications, in NCVs. We believe it is important for funders to align their open science policies or recommendations with what they are asking applicants to include in their NCV.

### Limitations

Our review only included templates written in English, which may have excluded NCVs from non-English-speaking countries. Additionally, our supplemental search strategy relied on healthresearchfunders.org, a health-focused resource. While this was the most comprehensive list available, it may have led to the exclusion of templates used by funders outside the health domain and of NCVs that might have been captured in a traditional search.

## Conclusion

This scoping review highlights several key insights. First, while the number of funders currently requiring NCVs remains low overall, federal granting agencies are among the early adopters. Second, some funders do not provide formal templates or explicit examples and guidance on how to complete narrative CV’s—this may create barriers for the researcher community as they confront this new CV format. Third, among the NCV templates identified, there are structural commonalities, and the possibility to streamline further to the extent that funders’ interests overlap. As interest in NCVs grows, addressing these gaps will be essential to realizing their potential as a fairer and more holistic tool for research assessment.

## Supporting information

S1. Appendix 1_Data management plan developed using the ‘open science’ template in the DMP Assistant

S2. Appendix 2_Grey Literature Search Approach

S3. Appendix 3_Screening and extraction forms

## Data Availability

The raw data and extended data (supplementary material) for this study, as well as the completed *PRISMA-ScR Reporting Checklist* ^24^ are available on the Open Science Framework ^23^ under a CC-By Attribution 4.0 International license (DOI: 10.17605/OSF.IO/4DGBR). ^34^
