# Supplementary material for: What goes in a funder’s Narrative CV?: A Scoping Review
Source: F1000Res. 2025 Dec 1;14:1345. [Version 1] doi: 10.12688/f1000research.170507.1 (PMC12820469; doi:10.12688/f1000research.170507.1)
Supplement: Peer review history

# Reviewer report: Reviewer response for version 1

**DOI:** 10.5256/f1000research.187975.r438583

**Published:** 2026-01-20

**Authors:** Noemie Aubert Bonn, Karen Stroobants

**Affiliations:** 1 Hasselt University, Hasselt, Belgium; 2 CultureBase Consulting, UK, UK

Thank you for the opportunity to review the manuscript “What goes in a funder’s Narrative CV?: A Scoping Review” submitted for publication at *F1000*.

 The manuscript is clearly presented, the methodology is rigorously reported, and the findings are useful both for meta researchers and for funders or organisations interested in implementing Narrative Curriculum Vitae (NCVs).

 To help the authors, we will address a few points we noted in the order they appear in the manuscript rather than in the order of importance. We mark these comment as (1) ‘major comment’, (2) ‘minor comment’, (3) ‘optional comment’, or (4) ‘small typo’ to identify whether we consider our comments to be (1) important to address and with a possible impact on the conclusions of the manuscript, (2) small and easy to address, with no real impact on the conclusions of the manuscript, (3) up to the author’s discretion on whether they wish to address this point or not, or (4) only a small typo that may need to be corrected, respectively. We hope our assessment will be useful to the authors.

Comments for the authors:

 Small typo - ABSTRACT – “First, number of funders currently requiring NCVs remains low overall” is missing ‘the’ to be ‘First, the number of…’

 Small typo - ABSTRACT – ‘CV’s’ is written with the apostrophe while it is written as CVs (or NCVs) throughout the rest of the manuscript

 Minor comment – In the introduction, the authors mention that “researchers have traditionally been evaluated based on quantitative metrics”, the use of the term ‘traditional’ is debated here. Metrics-based evaluations are mostly prominent since the 80’s, so it may be preferable to use a different term, such as ‘researchers are often evaluated based on….’ or ‘In the past few decades, researchers have mostly been evaluated….’ Etc.

 Minor comment – Still in the introduction, the authors mention ‘significant results’ a number of times. While we completely understand what they mean, it may be useful to specify, possibly in a parenthesis after the first instance, that these are meant as ‘statistically significant’ or as results what statistically align with the hypotheses tested’. This is a minor point, but one that may be relevant given the broad audience the manuscript may attract for whom significance may have a different meaning related to impact and importance rather than to statistical results.

 Small typo - Methods – Step 2 – “This manual search of funder websites involved using same three search terms” is missing ‘the’ before ‘same’

 Major comment – At Step 4 of the methodology, the authors mention that they calculated all percentages using a denominator of 21, yet 22 templates were included? Shouldn’t this be 22? We tested a few percentages and it seems like the authors used 22 — so this may only be a typo on this instance — but it needs clarifying.

 Major comment – Third paragraph of the Results section - The authors mention that the Finnish NCV was initially released in 2012. This surprised us for a number of reasons. First, given our interest in narrative CVs this work was especially relevant to us, and we knew about all the templates found except Finland. That piqued our curiosity and we looked up the Researcher’s Curriculum Vitae Template for Finland, which is indeed an interesting example. We would describe it as an itemised CV format with a prescriptive set of short-answer questions, much like the Canadian Common CV or some funders’ embedded online itemised CVs, but wonder why this was included as a narrative CV by the authors. The CV itself does not seem to mention the term ‘narrative’, it seems to ask about lists and also numbers and metrics. In addition, we are aware that the Federation of Finnish Learned Societies is considering developing a narrative CV template for use across Finland as this is not a measure that they themselves consider is implemented in Finland (meaning that it is unlikely they consider their template as a narrative CV). Second, 2012 is many years before the first discussions on narrative CVs I am aware of in Europe. The NIH Biosketch, which is often described as a precursor to NCV discussions in Europe, was only officially released in 2014, and the boom of NCV discussions in Europe started around 2019 with the Royal Society Résumé for Researchers ( https://royalsociety.org/blog/2019/10/research-culture/). We would therefore encourage the authors to add a bit more details about how they found this specific NCV and reflect on how its inclusion may have shaped their findings (e.g., high number of questions vs. others, more quantitative elements captured, request for total number of publications, total number of grants, request for full list of publications, etc.).

 Minor comment – In Table 3, the authors distinguish between several elements for which the distinction is not very clear. For example, in the Contributions to Open Science, the authors distinguish ‘General open science’, ‘Open research’ (the preferred term for Open Science in the UK to foster a more inclusive approach), and ‘Open Science initiatives’. The distinction between those is not entirely clear, and it would probably be helpful to merge these categories, especially since they are already merged in the text under “other open science aspects”. The category ‘Open source’ is also quite vague, do the authors mean ‘Open Source software and code’? Similarly, in the Contributions to broader society, the authors list ‘General societal impact(s)’ and ‘Social impact’, the distinction between those may be helped by adding a few examples if relevant.

 Optional comment – On page 10, the first paragraph after Table 3, the authors describe the elements specific to journal publications that are required from NCV templates. We would find it very interesting if they could also mention how many templates explicitly encouraged applicants to think beyond publications outputs. We leave this as optional as this is only a ‘wish list’ item for a question that is often asked in relation to NCVs, but it would not be fair to expect the authors to go back to their design to add this if it is not specifically their interest.

 Small typo - P.10, second paragraph – The authors use the term ‘dollar’. Given the diverse currencies covered by these templates, the term could be replaced by ‘sum of money awarded’ ‘amount of money awarded’ or something similar.

 Optional comment – From a user perspective, it would be really interesting to see an additional material file which includes all the questions form the narrative CVs included and organised by themes. This could be a useful first step to help bring a route for harmonisation in narrative cv templates, as supported by the authors.

 Small typo – Second paragraph of the Discussion – Reference [36] does not seem to have been added to the formatted references of the manuscript, it is also not present in the final reference list.

 Optional comment – On page 12, before-last paragraph, the authors describe the lack of clarity on how NCVs are being assessed based on what is described in the templates and instructions for applicants. They do not mention whether the funders made instructions for reviewers openly available. We understand that this is beyond the scope of the study, which is why we make this comment optional, but the authors could mention this caveat or explain if they saw any links to the instructions that reviewers receive. On this point, beyond the subjectivity that the lack of clarity on the assessment could have, the authors could also consider mentioning that it may create uncertainty with applicants, potentially encouraging them to stick to conventional outputs or making them feel a pressure to fulfil all the possible achievements that could be included in the narrative CV.

 Minor comment – On page 13, first line, the authors mention that “a coordinated approach would ease the burden on applicants”, we think they could also add ‘and reviewers’ to stress this even further.

 Minor comment – On page 13 (and related to a point above) – the authors bring back the point that “funders must be more transparent about how NCVs are evaluated. Clear criteria and reviewer guidelines would go a long way toward ensuring fair and consistent assessments. We would also like to see an evaluation of the effectiveness of any training administered to peer reviewers to demonstrate effectiveness.” Regarding this point, it would be important to know whether the authors searched for reviewer guidance or not, as this may influence this conclusion. If not, the authors should probably mention that the transparency of these criteria to applicants is what is considered here.

 Optional comment – In the discussion, it could be interesting if the authors discuss further the potential challenges in harmonising NCV templates. This is not necessary, but is an element that comes back frequently in discussions on NCV templates and seemingly one that adds complexity, especially between countries.

 Optional comment – In the conclusion, the authors mention that “while the number of funders currently requiring NCVs remains low overall, federal granting agencies are among the early adopters.” This point could be clarified by explaining that federal granting agencies generally have a greater reach and may have a bigger influence on practices from funders and institutions within the same country. Yet, this is a very minor point so we leave it entirely to the authors’ preference.

 Optional comment – In the conclusion, we would encourage the authors to bring back their point on the lack of clarity for reviewers’ instructions and guidance.

 We hope these small comments will be helpful to the authors. We are happy to be contacted for clarifications or further questions if needed.

Are the rationale for, and objectives of, the Systematic Review clearly stated?

Yes

Is the statistical analysis and its interpretation appropriate?

Partly

If this is a Living Systematic Review, is the ‘living’ method appropriate and is the search schedule clearly defined and justified? (‘Living Systematic Review’ or a variation of this term should be included in the title.)

Not applicable

Are sufficient details of the methods and analysis provided to allow replication by others?

Yes

Are the conclusions drawn adequately supported by the results presented in the review?

Yes

Reviewer Expertise:

Research assessment, research culture, open research, research integrity

We confirm that we have read this submission and believe that we have an appropriate level of expertise to confirm that it is of an acceptable scientific standard, however we have significant reservations, as outlined above.

# Reviewer report: Reviewer response for version 1

**DOI:** 10.5256/f1000research.187975.r441322

**Published:** 2026-01-06

**Authors:** Jamie Shaw

**Affiliations:** 1 Victoria College, University of Toronto Victoria College (Ringgold ID: 93777), Toronto, Ontario, Canada

Thank you for this helpful overview of current practices of NCVs, which will be useful for future research on how NCVs should be utilized. My comments are all very minor, as will become obvious.

 “First, number of funders currently” --> “First, the number of funders….”

 The first sentence could easily describe traditional CVs as well. Perhaps “Narrative Curriculum Vitae (NCVs) are a type of CV that use extended, structured prose explaining a researcher’s skills, experiences, collaborations, and achievements across a broad range of research activities”?

 “thus creating confusion and undermining the credibility of the NCV approach” – this is far too strong of a statement. Maybe “thus raising questions about the reliability of NCVs” or something like this.

 I am not sure that “subjectivity” is the right word here. Perhaps “idiosyncrasy” – subjectivity is about only being knowable to a particular subject, which is not at stake with NCVs.

 Another potential problem with NCVs is marginalizing non-native speakers.

 : Similarly, the total number of questions/items in the templates varied widely (range: 3-31 questions; median = 8.5 questions):

- Maybe a couple exemplar questions could be listed here?

“This burden is exacerbated by the lack of standardization across NCV formats, requiring applicants to tailor their CVs differently for each funder”

- This seems like a very minor issue, since the funding agencies are typically operating under different jurisdictions. It also seems a bit one-sided to call non-standardization “inefficient” since funding agencies require some tailoring for their needs. The recommendation to harmonize would need to account for their needs as well.

One thing that seems to deserve a bit of discussion is how the data on what is requested in narrative CVs reflects what is shifting from traditional CVs. Things like career breaks, as one example, are highly emphasized and not adequately addressed by traditional CVs. Talking a bit about the content of NCVs may be constructive to highlight what the practice is changing in terms of the information provided by applicants.

Are the rationale for, and objectives of, the Systematic Review clearly stated?

Yes

Is the statistical analysis and its interpretation appropriate?

I cannot comment. A qualified statistician is required.

If this is a Living Systematic Review, is the ‘living’ method appropriate and is the search schedule clearly defined and justified? (‘Living Systematic Review’ or a variation of this term should be included in the title.)

Not applicable

Are sufficient details of the methods and analysis provided to allow replication by others?

Yes

Are the conclusions drawn adequately supported by the results presented in the review?

Yes

Reviewer Expertise:

Philosophy of science

I confirm that I have read this submission and believe that I have an appropriate level of expertise to confirm that it is of an acceptable scientific standard.

# Reviewer report: Reviewer response for version 1

**DOI:** 10.5256/f1000research.187975.r441325

**Published:** 2026-01-05

**Authors:** Alejandra Recio Saucedo

**Affiliations:** 1 National Institute for Health and Care Research Coordinating Centre, Southampton, UK

The article *What goes in a funder’s Narrative CV?: A Scoping Review* presents an analysis of funding organisations that have implemented Narrative CVs. These are defined as CV formats focusing on written descriptions of researchers’ skills, experiences, collaborations, and achievements to promote more equitable and responsible research assessment.

 Overall, the description of the approach and methods used to design and conduct the review is clear and provides sufficient detail for replication. The publication of the protocol and supplemental materials strengthens open research practices and reduces research waste. The authors’ transition from thematic to content analysis is well-justified by the nature of the data collected. The aims of the review were successfully met, and the evidence collected directly addresses the two primary research questions:

1. Which research funding agencies currently provide NCV templates as part of their grant application process?
2. What are the characteristics of NCV templates provided by these agencies?

In the discussion of findings, the authors conclude with recommendations that have the potential to optimise the use of Narrative CVs in funding decisions and, importantly, reduce the administrative burden on applicants providing evidence of their expertise or transferable skills to contribute to research.

**General comments and clarifications:**

1. The authors define Narrative CVs as focusing on “written descriptions of researchers’ skills, experiences, collaborations, and achievements.” This definition raises a question regarding the core purpose of NCVs: Is the format intended to expand the types of outputs researchers use to evidence their skills, or is it aimed at encouraging professionals with diverse, non-research backgrounds to contribute via transferable expertise? Using the word “researchers” may inadvertently exclude essential members of the research ecosystem, including statisticians, software developers, and laboratory technicians, whose roles/careers may not follow traditional academic pathways.
2. While journal impact factors are often associated with prestige, the two are not synonymous. I suggest clarifying the phrasing: “...and the prestige, or ‘impact factor’, of the journals in which their research is published.”
3. I agree that research should be informed by a wide range of data and scientific designs. However, the assertion that a focus on “quantitative metrics can distort research priorities by shifting the focus away from meaningful scientific contributions onto high publication volumes and significant results” may be an overstatement. Meaningful contributions result from both quantitative and qualitative research; selecting one design over another depends on the research question/type of research/context and does not inherently affect the value of the research.
4. Could the authors clarify the statement: “NCVs permit assessments centered around the research subject”?
5. The authors state that “all percentages... were calculated using 21 NCV templates as the denominator.” However, the PRISMA flowchart indicates that 22 records were included, and the figures provided match a denominator of 22. This discrepancy should be addressed.

The authors highlight several urgent issues the sector must address that I list here to encourage further discussion:

**Variability in guidance available:** “Additionally, the degree of instruction varied widely—from minimal guidance to highly detailed examples... This variability may lead to confusion for applicants and inconsistencies in how reviewers interpret and assess NCVs.”

**Transparency in NCV assessment and use:** “A particularly concerning gap is the lack of transparency around the evaluation of NCVs... This lack of clarity introduces subjectivity, which could undermine both the credibility and consistency of NCV-based assessments. Increasing transparency is essential for standardizing expectations. Establishing a stakeholder committee could help address this shortcoming.”

**Effectiveness and impact of reviewer training:** An evaluation of peer reviewer training would be valuable: “We would also like to see an evaluation of the effectiveness of any training administered to peer reviewers to demonstrate effectiveness.”

Are the rationale for, and objectives of, the Systematic Review clearly stated?

Yes

Is the statistical analysis and its interpretation appropriate?

Yes

If this is a Living Systematic Review, is the ‘living’ method appropriate and is the search schedule clearly defined and justified? (‘Living Systematic Review’ or a variation of this term should be included in the title.)

Not applicable

Are sufficient details of the methods and analysis provided to allow replication by others?

Yes

Are the conclusions drawn adequately supported by the results presented in the review?

Yes

Reviewer Expertise:

Research on research

I confirm that I have read this submission and believe that I have an appropriate level of expertise to confirm that it is of an acceptable scientific standard, however I have significant reservations, as outlined above.
